# Editorial: Novel approaches for developing vaccines against influenza virus

**DOI:** 10.3389/fcimb.2024.1435768

**Published:** 2024-07-26

**Authors:** Rishi Kumar Jaiswal, Srijani Basu

**Affiliations:** ^1^ Department of Cancer Biology, Cardinal Bernardin Cancer Center, Loyola University Chicago Stritch School of Medicine, Maywood, IL, United States; ^2^ Naomi Berrie Diabetes Center, Columbia University, New York, NY, United States

**Keywords:** influenza, virus, vaccine, immune, modeling, SARS Cov2

The realm of vaccine research is continually evolving, with recent studies uncovering novel insights and proposing innovative approaches to combat infectious diseases effectively. These advances are critical in the face of emerging viral threats and the ongoing need for more efficient and broadly protective vaccines. This summary encapsulates key findings from four recent studies that explore different aspects of vaccine response, predictive modeling, and challenges in both human and animal vaccination. These insights not only enhance our understanding of the immune response mechanisms but also guide the development of next-generation vaccines. Here is a detailed consolidation of these pivotal topics in contemporary vaccine research (**Figure 1**).

**Figure 1:**
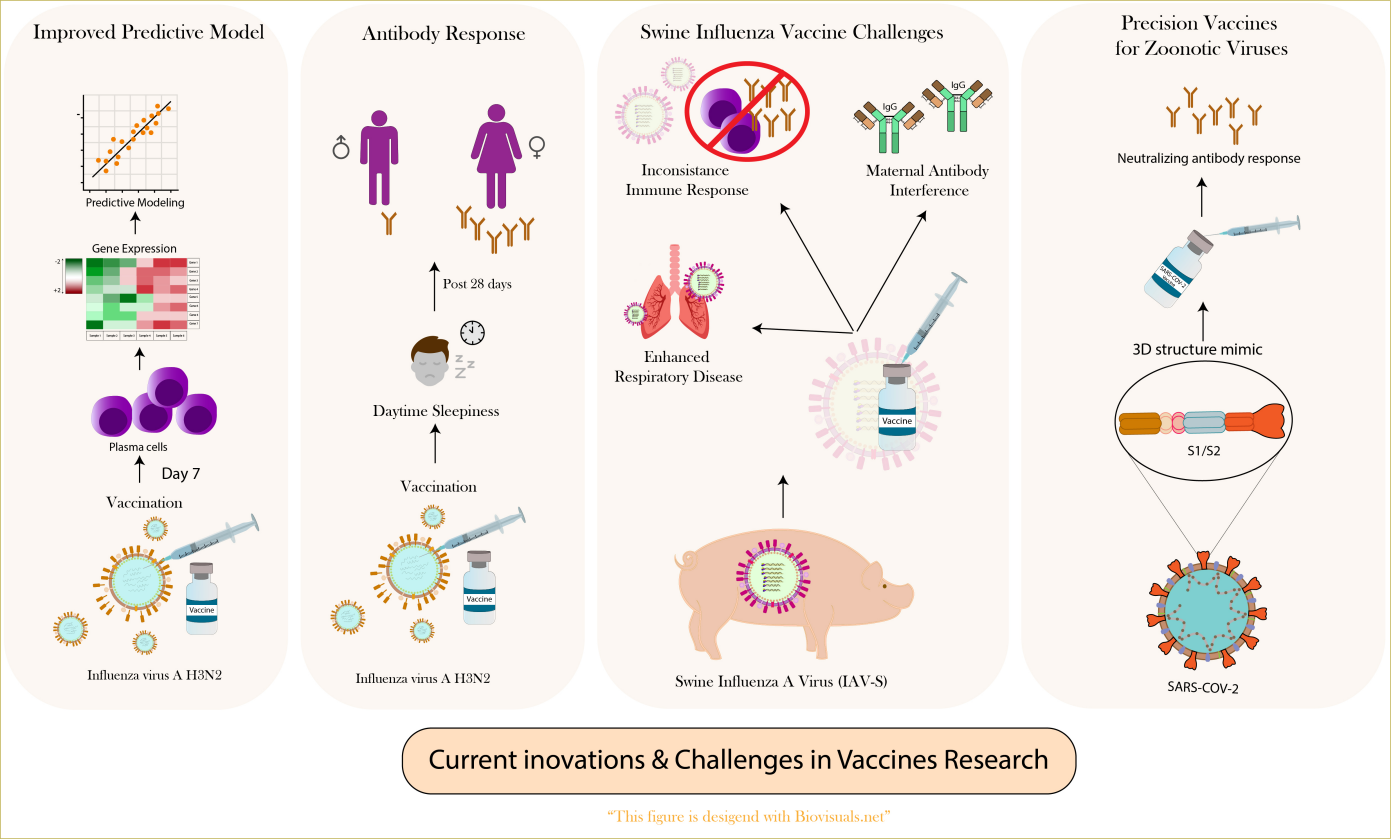
Comprehensive overview of innovations and challenges in vaccine research.

## Gender-specific vaccine response in humans

The study on influenza A/H3N2 vaccination revealed that excessive daytime sleepiness impairs the antibody response in male participants at Day 28 post-vaccination, but not in females. Quality of sleep and obstructive sleep apnea showed no significant correlation with antibody titers. This suggests a gender-specific interaction between sleep disruption and immune response to vaccination (Quach et al.).

## Predictive modeling of vaccine response to influenza

Research on predictive models for influenza vaccine response highlighted that models incorporating gene expression profiles from Day 7 post-vaccination performed optimally. Specifically, models based on differential fold change genes excelled in external validation. The inclusion of baseline predictors enhanced the models’ performance, particularly when the gene expression profile of plasma cells was used. This underscores the effectiveness of a combined modeling strategy leveraging single-cell level data for predicting vaccine efficacy (Ye et al.).

## Challenges in swine influenza vaccination

Swine Influenza A Virus (IAV-S) presents significant challenges to the pork industry and public health due to its rapid evolution and zoonotic potential. Current vaccines struggle with diversification of the virus, inconsistent immune responses, interference from maternal antibodies, and occasionally, enhanced respiratory diseases post-vaccination. Recent research has focused on developing universal vaccines that address these issues and improve the breadth of protective immune responses, while also exploring novel vaccine platforms to enhance control methods (Petro-Turnquist et al.).

## Advances in precision vaccines for zoonotic viruses

A novel approach in vaccine development against pathogenic zoonotic viruses, like SARS-CoV-2, focuses on proteolytic activation—a key mechanism for viral infectivity (Trabelsi et al.). By selecting specific immunogenic epitopes involved in this process and engineering candidate vaccines to mimic their native 3D structure, researchers have shown promising results. In studies involving COVID-19 patients and animal models, these engineered antigens induced a neutralizing antibody response, indicative of potential protective immunity. This method combines computational analysis with experimental biology to design precision vaccines effective across viral variants (Trabelsi et al.).

These studies collectively highlight innovative strategies and challenges in the field of vaccine research, emphasizing the importance of tailored approaches based on genetic, physiological, and epidemiological data to enhance vaccine efficacy and response prediction.

## Author contributions

RKJ: Writing – review & editing, Conceptualization, Formal Analysis. SB: Writing – review & editing, Writing – original draft.

